# Evolution of artificial ion channels

**DOI:** 10.1093/nsr/nwad293

**Published:** 2023-11-20

**Authors:** Yugang Wang

**Affiliations:** School of Physics, Peking University, China

Protein ion channels on a cell membrane generate action potentials for cellular signal transduction, and regulate biological activities during life. Since the discovery of the asymmetric ion transport phenomenon in quartz nanopipettes by Bard and colleagues [1], synthetic nanofluidic materials and devices have attracted a lot of research interests from the scientific community, with efforts having been made to mimic the ionic gating and electrogenic functions of biological ion channels [2]. Over the past two decades, this interdisciplinary field of physics, chemistry and materials science has developed artificial ion channels (AICs).

As human beings constantly gain fresh inspiration from mother nature, the structural and functional evolution of AICs never stops. For example, biological potassium ion channels are over three orders of magnitude more permeable for larger K^+^ (ionic radius ∼1.3 Å) than for smaller Na^+^ (∼1.0 Å) [3]. For decades, scientists have sought to realize this anti-size-exclusion selectivity with human-made materials, but very limited progress has been made in design and fabrication of artificial potassium channels with high K^+^/Na^+^ selectivity [4].

Writing in *National Science Review*, an international team led by Prof. Wei Guo at Beihang University addresses this problem by reassessing the fine structure of biological potassium ion channels (Fig. 1a) [5]. The selectivity filter is composed of four helical layers of carbonyl rings as binding sites for potassium ions. Interestingly, they find that the rotary carbonyl rings are a long-overlooked structural basis for the ultra-high K^+^/Na^+^ selectivity, and they design artificial potassium channels based on this feature.

**Figure 1.**
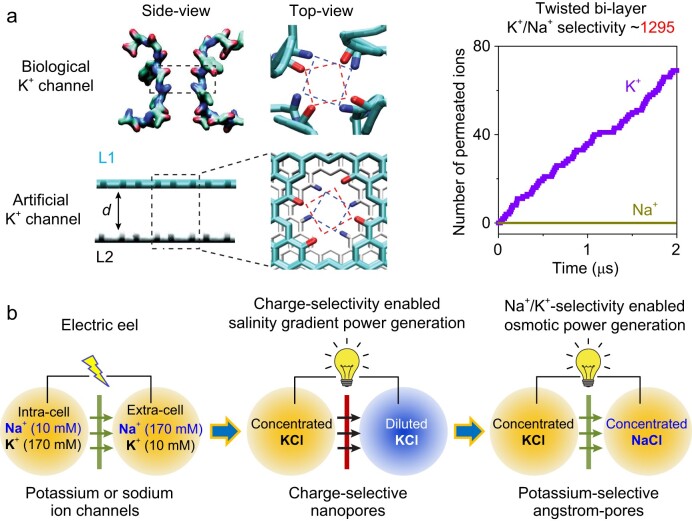
(a) Selectivity filter of a biological potassium ion channel. K^+^ binding sites exhibit a helical layered structure. Inspired by this feature, angstrom pores in a bilayer graphene membrane are chemically modified with twisted carbonyl groups on the pore edge. As an artificial potassium channel, the bilayer structure perfectly inhibits leaking of unwanted sodium ions. Reproduced with permission from ref. [5]. (b) Two generations of electric-eel-mimetic ionic power generation.

By computational means, the researchers innovatively fabricate angstrom-scale pores in a bilayer graphene membrane, and modify the carbon atoms on the pore edge with carbonyl groups on twisted positions (Fig. 1a). The designed AICs perfectly block the transport of non-specific Na^+^, and result in a record high K^+^/Na^+^ selectivity ratio of 1295. Atomic trajectories show that each K^+^ permeation event should involve at least two potassium ions confined in the angstrom pores. More intriguingly, an interlayer water molecule is found to play a key role in mediating the dual-ion transport process by forming a so-called K-H_2_O-K quasi-particle. As a proof-of-concept application, the potassium-specific AICs are proposed to harvest osmotic power by mixing ionic solutions of equal concentration, but with a different ionic composition of K^+^ and Na^+^ (they call it PoPee-OPG).

From a historical perspective, the use of AICs to mimic the discharge behavior of the electrocyte cells on the body of an electric eel has experienced two major generations of evolution (Fig. 1b). For the first generation, in a seminal work also contributed by Guo and colleagues [6], charge-selective nanopores were proposed to harvest salinity gradient energy by mixing ionic solutions of different concentrations. They established the first connection with the discharge principle of electric eels (Fig. 1b). By sacrificing a small amount of charge selectivity (10%–15%) relative to conventional ion-exchange membranes [7], the nanopore-based salinity gradient power generation (SGPG) greatly enhances the transmembrane ionic flux by one to two orders of magnitude, and thus promotes the overall power density >10 fold.

Now, PoPee-OPG represents a next-generation evolution (Fig. 1b). The total ionic strength across a biological cell membrane should be approximately equal to maintain a balance in osmotic pressure. PoPee-OPG eliminates the diluted ionic solution in previous SGPG. In this way, it greatly enhances the total concentration of ionic charge carriers, and thus enables high power.

More importantly, PoPee-OPG further shrinks the pore size from nanoscale to angstrom-scale, which is a fundamental requirement of ion-specific selectivity. The generated electrical power from a single angstrom pore might be trivial (∼0.13 pW), however, considering the extremely small pore size, a very high numeric density of 10^16^ pores/m^2^ can be achieved. Even so, the total porosity is still sufficiently low (<1%), and interference by concentration polarization can be safely ignored [8]. Therefore, kilowatt-scale ionic power generation can be expected by exploiting parallelization.

It is encouraging to know that the twisted pore structure plays a key role in the precise sieving of molecular components with very close kinetic size, such as CO_2_ and C_2_H_2_ [9]. It is also worth noting that the metastable K-H_2_O-K quasi-particles can hold the two like-charged potassium ions to a tiny distance of no more than 4 Å, exhibiting a weak mutual attraction between the confined cations. This feature is reminiscent of the paired electrons in BCS theory (as applied to superconductors), but the bound electrons have a much longer extension of several microns [10]. Short coherence length means better resistance to thermal perturbations. Therefore, one can put forward a hypothesis that the confined ionic pairs may have implications for future room-temperature superconducting materials using ions as charge carriers.

## References

[1] Wei C , Bard A, Feldberg S. Anal Chem 1997; 69: 4627–33. 10.1021/ac970551g

[2] Guo W , Tian Y, Jiang L. Acc Chem Res 2013; 46: 2834–46. 10.1021/ar400024p 23713693

[3] Gouaux E , Mackinnon R. Science 2005; 310: 1461–5. 10.1126/science.1113666 16322449

[4] Zhang H , Li X, Hou J et al. Chem Soc Rev 2022; 51: 2224–54. 10.1039/D1CS00582K 35225300

[5] Li J , Du L, Kong X et al. Natl Sci Rev 2023; 10: nwad260. 10.1093/nsr/nwad260 37954195 PMC10632797

[6] Guo W , Cao L, Xia J et al. Adv Funct Mater 2010; 20: 1339–44. 10.1002/adfm.200902312

[7] Logan B , Elimelech M. Nature 2012; 488: 313–9. 10.1038/nature11477 22895336

[8] Cao L , Wen Q, Feng Y et al. Adv Funct Mater 2018; 28: 1804189. 10.1002/adfm.201804189

[9] Zhu H , Xue W, Huang H et al. Sci Bull 2023; 68: 2531–5. 10.1016/j.scib.2023.09.008 37739844

[10] Cooper L. Phys Rev 1956; 104: 1189–90. 10.1103/PhysRev.104.1189

